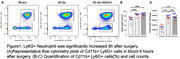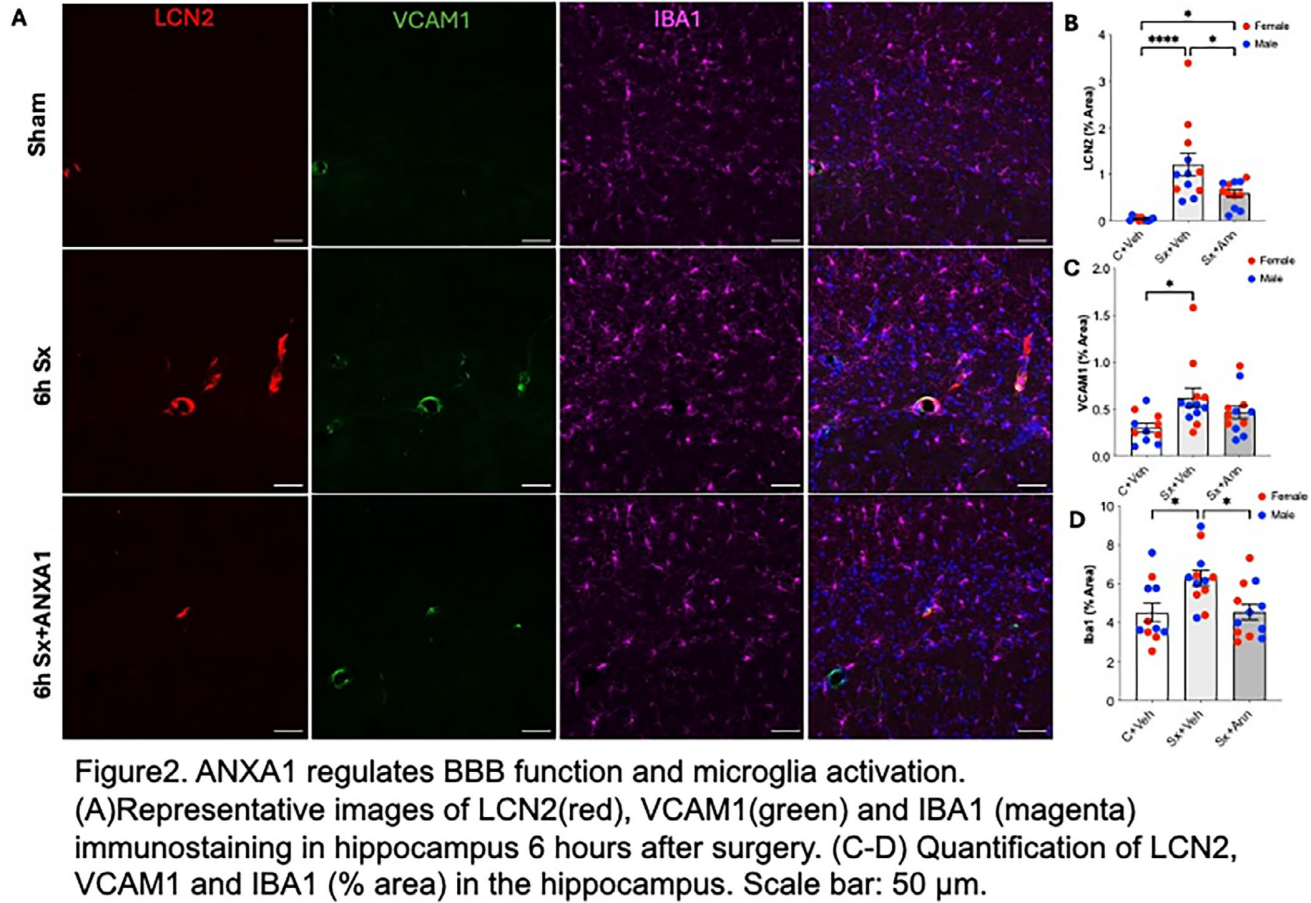# Annexin‐A1 improves neurovascular pathology in delirium superimposed on dementia

**DOI:** 10.1002/alz70855_100910

**Published:** 2025-12-23

**Authors:** Chengcheng Song, Ting Yang, Niccolo Terrando

**Affiliations:** ^1^ Duke University School of Medicine, Durham, NC, USA

## Abstract

**Background:**

Postoperative delirium is a significant problem in older adults, especially in subjects with underlying neurodegeneration. Peripheral surgical trauma has been described as a trigger for microglial activation and cognitive deficits, with the blood‐brain barrier (BBB) being implicated in the pathophysiology of delirium both in preclinical and clinical studies. However, no therapies are currently available to treat delirium superimposed on dementia (DSD).

**Method:**

We tested the neuroprotective effects of Annexin‐A1 (ANXA1) using the 12‐month old 3XTg‐AD mouse model following orthopedic surgery. The expression of Ly6G^+^ neutrophil was detected by flow cytometry. Endothelia markers, fibrinogen and VCAM1 were investigated after surgery. Microglial activation was also evaluated.

**Result:**

Orthopedic surgery induced a robust inflammatory response in 3XTg mice both at 6 hr with neutrophilia. Treatment with ANXA1 did not impact the number of circulating neutrophils in the plasma but significantly reduced BBB dysfunction (including expression of fibrinogen and VCAM1) and microglial activation (IBA‐1 and CD68).

**Conclusion:**

ANXA1 could be a new therapy targeted to protect the BBB in DSD.